# Demosaiced pixel super-resolution for multiplexed holographic color imaging

**DOI:** 10.1038/srep28601

**Published:** 2016-06-29

**Authors:** Yichen Wu, Yibo Zhang, Wei Luo, Aydogan Ozcan

**Affiliations:** 1 Electrical Engineering Department, University of California, Los Angeles, CA, 90095, USA; 2 Bioengineering Department, University of California, Los Angeles, CA, 90095, USA; 3 California NanoSystems Institute (CNSI), University of California, Los Angeles, CA, 90095, USA; 4 Department of Surgery, David Geffen School of Medicine, University of California, Los Angeles, CA, 90095, USA

## Abstract

To synthesize a holographic color image, one can sequentially take three holograms at different wavelengths, e.g., at red (R), green (G) and blue (B) parts of the spectrum, and digitally merge them. To speed up the imaging process by a factor of three, a Bayer color sensor-chip can also be used to demultiplex three wavelengths that *simultaneously* illuminate the sample and digitally retrieve individual set of holograms using the known transmission spectra of the Bayer color filters. However, because the pixels of different channels (R, G, B) on a Bayer color sensor are not at the same physical location, conventional demosaicing techniques generate color artifacts in holographic imaging using simultaneous multi-wavelength illumination. Here we demonstrate that pixel super-resolution can be merged into the color de-multiplexing process to significantly suppress the artifacts in wavelength-multiplexed holographic color imaging. This new approach, termed Demosaiced Pixel Super-Resolution (D-PSR), generates color images that are similar in performance to sequential illumination at three wavelengths, and therefore improves the speed of holographic color imaging by 3-fold. D-PSR method is broadly applicable to holographic microscopy applications, where high-resolution imaging and multi-wavelength illumination are desired.

Computational microscopy modalities are becoming more and more powerful thanks to the rapid improvements in digital imaging chips, graphics processing units as well as emerging image reconstruction methods that enable high-resolution imaging over large sample areas and volumes^1^,^2^,^3^,^4^,^5^,^6^,^7^,^8^,^9^,^10^,^11^,^12^,^13^,^14^,^15^,^16^,^17^,^18^,^19^. Among these different computational microscopy techniques, digital holography is one of the most widely explored modalities as it permits high-throughput 3D imaging of phase and amplitude information of specimen^20^,^21^,^22^,^23^,^24^,^25^,^26^,^27^. Holographic microscopy in general demands spatial and temporal coherence of illumination, although partially-coherent or even incoherent sources can also be utilized in certain imaging designs. To achieve color imaging in digital holography various methods have been employed^2^,^25^,^28^,^29^,^30^,^31^,^32^,^33^,^34^. One of the most commonly used approaches sequentially captures three holograms at different wavelengths, at red (e.g., 610–650 nm), green (e.g., 520–560 nm) and blue (e.g., 450–480 nm) parts of the spectrum, and then digitally cross-registers and combines these holograms to reconstruct a color image of the specimen^25^,^29^,^30^,^31^,^33^,^34^.

As an alternative to this sequential color illumination method, *simultaneous* multi-wavelength illumination of the sample has also been utilized in combination with a color imager chip (e.g., with a Bayer color-filter array, CFA) to digitize the resulting multi-color hologram in one snap-shot^29^,^30^. Using the known transmission spectra of the red (R), green (G) and blue (B) filters of the Bayer CFA, three sets of holograms corresponding to three unique wavelengths can be digitally retrieved through an inverse mapping (i.e., de-multiplexing) algorithm^29^. Compared to sequential color illumination, this simultaneous illumination approach saves experimental time through digital de-multiplexing of color channels; however, the reconstructed color images are lower resolution and exhibit color artifacts. Unlike natural images, holograms contain rapidly changing oscillations/fringes and since different channels of the color filters of a Bayer pattern are not exactly at the same spatial location, the traditional Bayer demosaicing process, when dealing with the sharp oscillations of a hologram, causes severe fringe artifacts^29^,^30^,^35^,^36^, which become even more noticeable for wide-field holographic imaging systems with large effective pixels or small magnifications. To better handle such sampling artifacts, different Bayer demosaicing approaches have also been proposed^35^,^36^,^37^,^38^,^39^, however, these methods are still short of creating an artifact-free de-multiplexing of holographic high frequency fringes created by multi-wavelength illumination.

To tackle these sampling and de-multiplexing related challenges in holographic color imaging, here we introduce a new high-resolution color microscopy technique termed Demosaiced Pixel Super-Resolution (D-PSR). In this D-PSR approach, we first capture a set of raw holograms on a Bayer color sensor chip using *simultaneous* multi-wavelength illumination, where the sensor plane is shifted by small increments. We then perform pixel super-resolution based on these sub-pixel shifted raw holograms^40^,^41^ to digitally synthesize smaller pixels (e.g., by a factor of ~3 fold) for each element of the Bayer CFA. Using the pre-calibrated spectral cross-talk matrix of each filter of the Bayer CFA at our selected illumination wavelengths, we de-multiplex three color channels, each of which is also pixel super-resolved. As will be demonstrated in our Results section, this D-PSR approach solves Bayer CFA related spatial sampling limitations and color artifacts of previous color de-multiplexing approaches, significantly improving the performance of holographic high-resolution color imaging.

For experimental demonstration of our D-PSR approach we selected lens-free holographic on-chip imaging, where the sample is placed on the top of a Bayer image sensor chip, typically at a distance of ~0.3–1 mm away from the chip surface. In this unit magnification transmission imaging set-up on a chip, the sample field-of-view (FOV) is equal to the active area of the sensor chip, which is typically ~20–30 mm^2^ using a state-of-the-art CMOS imager chip. As a result of this unique imaging configuration, the FOV and resolution are decoupled from each other and partially coherent sources can be utilized to push the resolution of the reconstructed holograms to the diffraction limit^27^,^34^,^42^,^43^. Another important advantage of this on-chip holographic imaging approach is the compactness and cost-effectiveness of its set-up, which makes it highly suitable for telemedicine applications and field use. Since this is an in-line holographic imaging geometry, the twin-image noise that is characteristic of an in-line set-up needs to be eliminated, and we used multi-height based phase retrieval^2^,^44^,^45^ for this purpose. Through these on-chip microscopy experiments, we demonstrated that D-PSR achieves a color imaging performance that is comparable to sequential illumination of the sample at three distinct wavelengths (corresponding to R, G and B channels) and therefore improves the overall speed of holographic color imaging. Finally we emphasize that this D-PSR technique is broadly applicable to any holographic microscopy application (lens-based or lens-free), where high-resolution imaging and simultaneous multi-wavelength illumination are sought.

## Methods

### Optical setup and data acquisition

**Figure 1:**
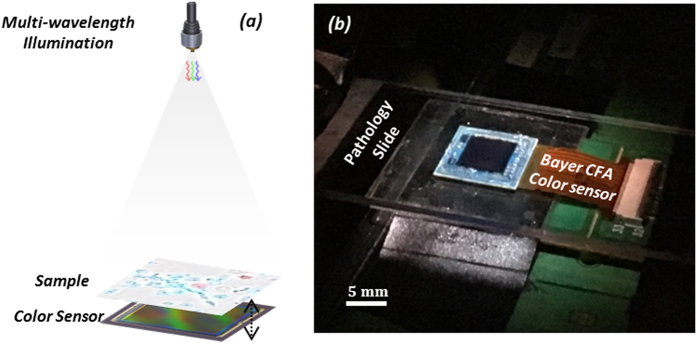
Wavelength-multiplexed holographic color imaging setup. (**a**) Schematics of the set-up. The sample (e.g., a pathology slide) is placed ~6 cm below the illumination fiber aperture and its in-line transmission hologram is captured by a Bayer color sensor chip that is placed at <1 mm below the sample. The sensor is mounted on a mechanical stage for 3D motion. (**b**) Photo of the same on-chip holographic imaging set-up.

As shown in Fig. 1, we use an in-line holographic lens-free on-chip imaging geometry. A broadband source (WhiteLase-Micro; Fianium Ltd, Southampton, UK) is filtered by an acousto-optic tunable filter down to ~5 nm bandwidth and is coupled to a single mode fiber to generate partially-coherent illumination across the field-of-view (~20 mm^2^) of our lens-free imaging setup. This source can simultaneously output up to 8 wavelength channels into the same fiber optic cable, and we used three spectral bands in our experiments (i.e., ~470 nm, ~530 nm, ~625–630 nm) to create multi-color illumination. This multiplexed partially coherent light, coming out of the fiber optic cable, propagates ~6 cm, and impinges on the specimen plane. Each of the three wavelength channels is partially diffracted by the sample and generates three independent in-line holograms to be sampled by a Bayer color CMOS sensor (16.4 Mpixel, 1.12 μm pixel size, Sony Corp., Japan), which is placed ~0.4 mm below the sample plane. The pixels of this color CMOS imager chip have four channels, namely B (Blue), G1 (Green 1), G2 (Green 2), and R (Red), which form a 2 × 2 period of the Bayer pattern on the imager chip. These filters have different transmission spectra, which will be detailed later on, and this information is crucial for spectral de-multiplexing of the acquired holograms. The color CMOS sensor chip is also mounted on a computer controlled 3D motion stage to permit: (1) lateral sub-pixel shifts between the imager chip and the sample hologram which is used to generate pixel super-resolved holograms, and (2) axial modulation of the sample-to-sensor distance which is used for multi-height based phase retrieval (i.e., twin-image elimination). The entire data acquisition process is automated by a custom-developed LabVIEW program.

### Pixel super-resolution

**Figure 2:**
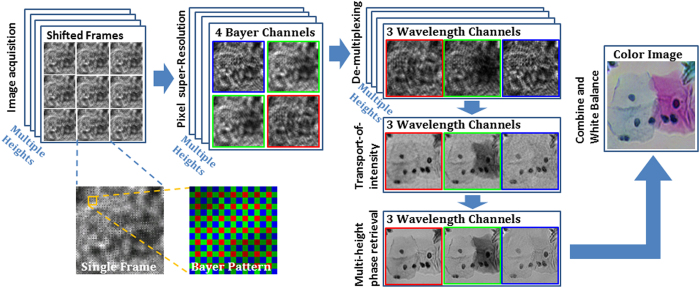
Computational imaging flow-chart of D-PSR method.

The reconstruction algorithm of D-PSR (Fig. 2) starts with the implementation of pixel super-resolution. Pixel super-resolution is a technique that deals with the spatial under-sampling problem in an imaging system^40^,^41^, in which a series of sub-pixel shifted low resolution images are acquired to digitally synthesize a high resolution image of the object, significantly increasing the space-bandwidth product of the imaging system^27^. In our experiments, to achieve pixel super-resolution, the stage was programmed to move the sensor laterally on a 6 × 6 grid and at each grid point a low-resolution raw hologram is captured. Each recorded raw hologram intensity is then separated into four Bayer channels (namely B, G1, G2, and R) and for each one of these channels, we used a conjugate-gradient-based pixel super-resolution method^40^,^41^ to synthesize a super-resolved hologram with an effective pixel size of ~0.37 μm at each sample-to-sensor height. The spatial location of each channel with respect to the others is also taken into account and digitally corrected for; therefore this pixel-super resolution step enables all the Bayer channels (B, G1, G2 and R) to be virtually super-imposed onto each other, which is important to mitigate the artifacts in the subsequent demosaicing steps.

### De-multiplexing of pixel super-resolved holograms

**Figure 3:**
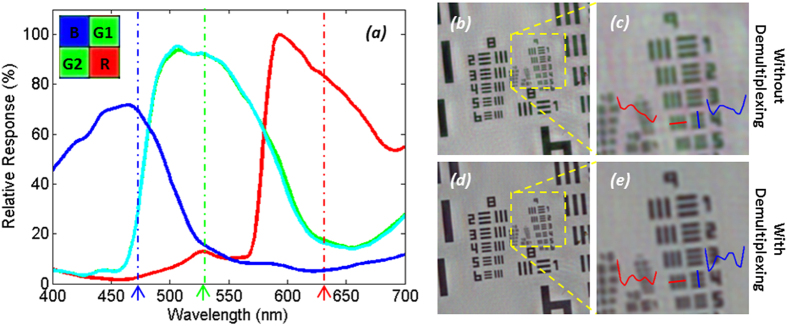
Spectral cross-talk in wavelength-multiplexed color imaging. (**a**) Measured spectra of B, G1, G2 and R channels of our Bayer sensor, showing ~15% cross-talk at the multiplexed wavelengths in our experiments (marked by dashed lines, also see Table 1). (**b,c**) Reconstruction of a resolution test chart using wavelength-multiplexed illumination with pixel super-resolution *without* the de-multiplexing step. Significant distortions in high-resolution features are observed, as also shown with red and blue highlighted cross-sections. (**d**,**e**) Reconstruction of the same data set *with* digital de-multiplexing. Previously unresolvable spatial features can now be resolved.

The transmission spectra of the four Bayer channels on a color CMOS imager contain considerable color cross-talk among channels (see e.g., Fig. 3(a)). For each pixel of the imager chip, we can formulate this spectral cross-talk as a matrix (*W*), such that:


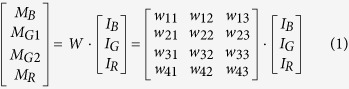


where *M*_*B*_, *M*_*G*1_, *M*_*G*2_, *and M*_*R*_ correspond to the pixel super-resolved intensity values for each channel (i.e., the output of the previous sub-section), and *I*_*B*_ *I*_*G*_ *and I*_*R*_ refer to the de-multiplexed holograms corresponding to the three illumination wavelengths, before the spectral mixing occurred at the color sensor chip. The entries of the cross-talk matrix 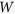 are determined by the transmission spectra of the Bayer CFA. Although the transmission spectrum of each Bayer filter is usually provided by the manufacturer of the sensor-array, we experimentally calibrated it to get more accurate results. For this purpose, we first recorded the background (i.e., object-free) response of the sensor chip from 400 nm to 700 nm at 5 nm steps. A 400-by-400 pixel region at the center of the sensor chip is averaged for each channel, and after normalization of the illumination power at each wavelength, measured using a power-meter (Thorlabs PM100, S120UV sensor head), the resulting curve for each channel is then taken as the spectral response of each Bayer filter on the imager chip (see e.g., Fig. 3(a)). We should emphasize that for a given color imager chip, these spectral cross-talk calibration curves need to be measured *only once*. Based on these measured spectra, the cross-talk matrix (*W* in Eq. (1) can be inferred for any arbitrary set/choice of illumination wavelengths that are multiplexed in our holographic color imaging experiments (see e.g., Table 1).

Based on this spectral cross-talk matrix, the de-multiplexed holograms corresponding to the three simultaneous illumination wavelengths in our holographic imaging set-up can then be determined through a *left inverse* operation:


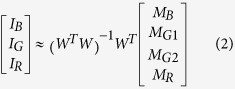


where the superscript *-1* refers to the inverse and *T* refers to the transpose of a matrix.

### Multi-height based phase retrieval

One drawback of in-line holographic imaging geometry is its twin image noise. Additional constraints, such as the object support^46^, sparsity^5^,^47^,^48^ or multiple measurements at different heights^44^ or illumination angles^34^ can be employed to eliminate the twin image noise. For spatially dense and connected objects we usually utilize multi-height based phase retrieval because it is relatively hard to define an object support for such connected samples. In this multi-height based iterative phase retrieval algorithm^2^,^44^,^45^, we start from one of the pixel super-resolved holograms and digitally propagate it to the next measurement height, where we replace the amplitude of the field with the measured amplitude, and then propagate it to the next height until we reach the last measurement. The same process is repeated backward and then forward for e.g., 20–30 iterations. Each wave propagation operation is done using the angular spectrum method^49^. For faster convergence, we also use the solution to the transport-of-intensity equation (TIE) as our initial phase guess for multi-height phase retrieval^2^,^50^. In the experiments reported in this manuscript, we measured holograms at 4 consecutive heights that are axially separated by ~30 μm.

### Saturation correction in digitization of wavelength-multiplexed holograms

When using the D-PSR approach for imaging of biological samples, a saturation-related de-multiplexing color artifact can sometimes be observed, as also illustrated in Supplementary Fig. S1. Although pixel saturation can be avoided by reducing the exposure time to a point where no pixels are saturated, this will result in unacceptable loss of information, as most of the pixels will then use only a small portion of the dynamic range. Alternatively, here we use a Bayesian-estimation-based saturation correction algorithm^51^, which uses the unsaturated pixels from other color channels at the same physical location to get a better estimate of the saturated pixels. It is theoretically proven that, using this Bayesian estimation approach, the corrected image will always have a smaller error than the uncorrected saturated one^52^.

Below, we will detail the use of this saturation correction method in our D-PSR approach. We assume that for a given raw image, the pixel values of different color channels follow a normal distribution:


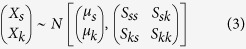


where 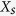 and 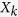 denote pixel values of saturated and unsaturated channels, 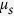 and 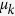 represent their mean, respectively, and 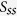, 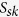, 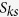 and 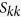 represent their covariance. The saturated channel 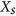 can be replaced by its statistical expectation, using the known non-saturated channel measurements 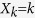 at the same pixel location:


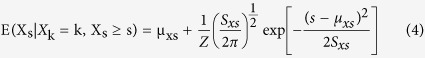


where:


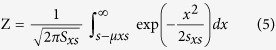



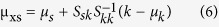



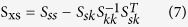


Note that since the spectral response of G1 and G2 channels are nearly identical, we took the average of these two super-resolved channels and treated it as the same channel G - *only* for this saturation correction step. We numerically implemented this saturation correction algorithm (see Supplementary Fig. S2) in five steps, as follows:

***Step 1***. Estimate the a-priori mean 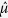 and co-variance 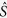 of the unsaturated pixel values of R, G and B channels:


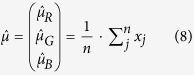



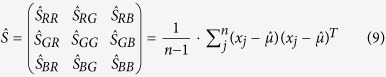


where 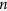 is the total number of un-saturated pixels in the image, 
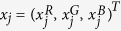
 is a vector that represents the pixel values of R, G, B channels at pixel location 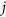.

***Step 2***. After defining a saturation level *s*, the distance 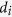 of all the channels 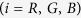 to the saturation level (*s*) can be determined as:


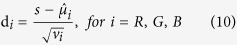


where 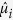 and 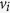 define the mean and the variance of all the unsaturated pixels in color channel 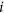, respectively. We typically choose *s* = 1020 for our 10 bit depth image sensor.

***Step 3***. Start from the most saturated channel, i.e. the channel 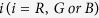 that has the smallest distance 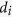 to the saturation level, and replace the values of these saturated pixels with the expectation value calculated using Eq. (4). All the pixels in the other two un-corrected channels are taken as valid pixels.

***Step 4.*** Correct the second most saturated channel 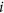 using Eq. (4), taking the corrected most saturated channel and the other un-corrected channel as valid pixels.

***Step 5.*** Correct the third (last) saturated channel using Eq. (4), taking the corrected values of the first and the second most saturated channels as valid pixels.

We typically run Steps 3–5 iteratively (e.g., for 3 iterations) to get improved results. As illustrated in Supplementary Fig. S1, the de-multiplexing color artifacts shown in the first column are greatly alleviated with this additional saturation correction step (second column), resulting in a reconstructed color image that is similar to a sequentially taken RGB image (third column).

### White-balancing of wavelength-multiplexed holograms

Although the power levels of the multiplexed illumination wavelengths during our measurements are adjusted so that their detected intensities are very close to each other, there are still small uncontrolled variations among color channels. To correct for these power variations, we first choose a uniform background (empty) region of the captured hologram, calculate the average of each Bayer channel within this selected region which is taken as the relative power level of each illumination wavelength. All the reconstructed holographic images are then normalized using these calculated power ratios to get a white-balanced image.

### Optimization of the choice of illumination wavelengths in D-PSR

Typically three illumination wavelengths are multiplexed in our D-PSR experiments, which are assigned to B, G and R channels, respectively. In this work, we also addressed the following question: *if one could arbitrarily choose these three illumination wavelengths, what would be the optimal wavelength range for each source to be multiplexed*? Intuitively, the optimality of the selected wavelengths depends on the transmission spectra (i.e., wavelength cross talk) of the color filters on the imager chip, as well as the transmission characteristics of the specimen to be imaged. Since we aim here general purpose microscopic imaging, we do not consider optimization of the illumination as a function of the sample spectral characteristics, and therefore only consider the transmission spectra of the CFA on the imager chip.

If the multiplexed channels are chosen to be too close in wavelength, the cross-talk among them will be too strong, and the illumination power of one or more channels needs to be reduced to accommodate the finite bit-depth of the digital sensor, which in turn will cause loss of spatial information. To better understand how this de-multiplexing error varies according to the selection of the multiplexed illumination wavelengths, we conducted a brute-force search of all the possible wavelength combinations for the spectral range of 400 nm to 700 nm with 1 nm step size and compared the resulting de-multiplexing errors (see the Supplementary Information and Supplementary Fig. S3 for further details). As illustrated in Supplementary Fig. S4, the differences among the de-multiplexing errors for different wavelength combinations are smaller than 6% over a large spectral range (>50 nm), which also contains the typical choice of red (610–650 nm), green (520–560 nm) and blue (450–480 nm) illumination bands. Based on this, we concluded that for a typical Bayer sensor, like the sensor we used in this work, the range of wavelength combinations that can be used for simultaneous illumination of the sample is rather large.

## Results and Discussion

**Figure 4:**
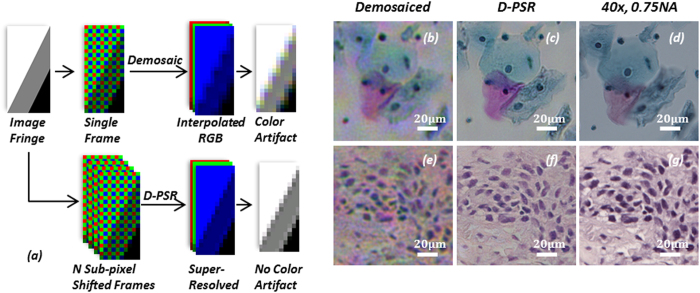
Comparison of D-PSR against conventional demosaicing for wavelength-multiplexed digital holographic color imaging. (**a**) Conventional demosaicing generates color artifacts at holographic fringes, which are avoided in D-PSR. (**b**,**e**) Multi-height phase retrieval based hologram reconstruction from 4 heights using conventional demosaicing. A single hologram is captured under multi-wavelength illumination at each height. (**c**,**f**) Multi-height phase retrieval based hologram reconstruction from 4 heights using *D-PSR*. 6 × 6 sub-pixel shifted holograms are captured under multi-wavelength illumination at each height. (**d**,**g**) Microscope images of the same regions of interest using a 40×, 0.75 NA objective-lens. (**b**–**d**) correspond to a stained Pap smear sample. (**e**–**g**) correspond to a stained breast cancer tissue sample. Bilinear demosaicing is used in (**a**,**b**,**e**).

When we multiplex the illumination wavelengths and simultaneously record the resulting holograms using a Bayer imager chip, there will be mainly two types of artifacts generated: (1) the spectral cross-talk among different Bayer filters will create pixel level mixing of holographic information of different illumination wavelengths (see e.g. Fig. 3(a)); and (2) spatial demosaicing artifacts will be created because the Bayer mosaicing geometry has 4 color channels (B, G1, G2, and R) that are spatially separated by one pixel shift, and requires the interpolation of neighboring pixels for the missing spatial information, which gives rise to fringe artifacts in holograms (see Fig. 4(a)). Conventional demosaicing techniques^35^,^53^ employed in digital cameras and photography literature rarely suffer from these artifacts as most natural images are spatially smooth. However, when dealing with multi-color digital holographic microscopy, the recorded holograms contain rapid oscillations and fringes, and therefore using a conventional demosaicing approach will result in severe color artifacts.

The first problem listed above, i.e., the spectral cross-talk issue, can generate strong high-frequency artifacts if left uncorrected. Experimental examples of these artifacts are illustrated in Fig. 3(b,c), where we show *pixel super-resolved* holographic image reconstructions *without* de-multiplexing and compare them against our D-PSR results (Fig. 3(d,e)), which show significant improvements especially in high-resolution features. As detailed in our Methods section, this issue can be tackled by digital de-multiplexing (through Eq. (2)). However, if we perform this de-multiplexing step directly on demosaiced Bayer pixels (i.e., *without pixel super-resolution*), it will also generate color artifacts for holographic imaging at interpolated fringes (e.g., see Fig. 4(a)), and such fringe artifacts at the hologram plane will spread out to the whole reconstructed image and generate severe rainbow artifacts, as can be seen in Fig. 4(b,e). D-PSR results for the same samples (Fig. 4(c,f)) show significant improvements and suppression of such color artifacts, in addition to having much better spatial resolution compared to interpolation-based de-multiplexing results shown in Fig. 4(b,e).

**Figure 5:**
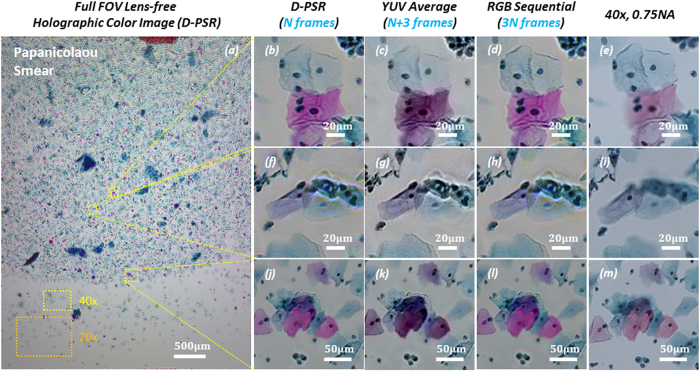
Wide field-of-view lens-free holographic color imaging of a Papanicolaou Smear. (**a**) Full field-of-view lens-free holographic image that is reconstructed using D-PSR under wavelength-multiplexed illumination at 470 nm, 527 nm and 624 nm. N = 144 raw holograms are used for this reconstruction. (**b**,**f**,**j**) Zoomed-in regions of (**a**). (**c**,**g**,**k**) Same zoomed in regions-of-interest reconstructed using the YUV color-space averaging method^32^; N+3 = 147 raw holograms are used. YUV color-space averaging method shows intensity bias and color leakage artifacts. (**d**,**h**,**l**) Same zoomed in regions-of-interest reconstructed using sequential RGB illumination^34^; 3N = 432 raw holograms are used. (**e**,**i**,**m**) Lens-based microscope images of the same samples are provided for comparison. These microscope images are blurred in some regions due to limited depth-of-focus of the objective-lens compared to lens-free holographic imaging. Typical FOVs of a 40× and a 20× objective-lens are also shown in (**a**).

Next, we imaged color-stained Papanicolaou smears (Pap smears) that are frequently used for screening of cervical cancer in order to compare the color imaging performance of D-PSR against some of the previously reported holographic color imaging techniques, including sequential RGB imaging^29^,^34^ and YUV color-space averaging^32^. As illustrated in the experimental comparison that is provided in Fig. 5, D-PSR has a very similar color imaging performance compared to sequential RGB imaging; however, by benefiting from simultaneous multi-wavelength illumination, D-PSR uses 3 fold less number of measurements compared to sequential color imaging, which makes it much more data efficient and faster. YUV color-space averaging, on the other hand, acquires a similar number of raw measurements/holograms compared to D-PSR, i.e., N + 3 vs. N, respectively, where N is the number of raw measurements that D-PSR uses. But the color imaging performance of YUV color-space averaging technique is inferior to D-PSR as it shows color bias and artifacts, also causing color leakage at the borders of rapid spatial transitions as illustrated in Fig. 5(c,g,k). In the last column of Fig. 5, microscopic images of the same sample regions of interest taken using a 40× 0.75 NA objective-lens are also shown for comparison. Note that the lens-based microscope images are blurred in some regions because of the limited depth-of-focus compared to lens-free microscopy images. Furthermore, to emphasize the large FOV advantage of lens-free on-chip microscopy, typical FOVs of 40× and 20× objective-lenses are also shown in Fig. 5(a).

We should also note that, in addition to 3 fold imaging speed improvement and reduced number of measurements compared to sequential color illumination, there are other reasons that sometimes simultaneous multi-wavelength illumination is preferred and D-PSR could be applied. For example, in imaging flow-cytometry systems, specimens (e.g., parasites or cells of interest) are constantly moving in a flow, and a motion-based PSR approach^54^ can be combined with D-PSR to get color images of the flowing micro-objects without the need for sequential multi-color illumination, which would directly improve the flow rate and the throughput of the imaging cytometer.

Finally, it is important to emphasize that the use a color (e.g., a Bayer RGB) sensor-chip, as compared to a monochrome sensor, has several advantages for holographic microscopy applications. First, color imagers are much more cost-effective compared to their monochrome versions due to economies of scale and their massive volume in consumer electronics market, especially in mobile-phones. Second, most of these small pixel pitch CMOS imager chips, including the one that is used in this work with ~1.1 μm pixel size, are not available for sale in monochrome format, which limits the spatial resolution that one can achieve using on-chip microscopy techniques with a monochrome chip. And third, the Bayer CFA of a color imager chip provides an excellent framework for multiplexed high-resolution color imaging of specimen as demonstrated in this manuscript.

## Conclusion

We demonstrated a new holographic color imaging technique that is termed Demosaiced Pixel Super-Resolution (D-PSR) that merges Bayer demosaicing and pixel super-resolution steps to achieve wavelength-multiplexed holographic color imaging. Demosaicing induced holographic color artifacts that arise due to limited spatial sampling at a Bayer CFA are significantly alleviated in D-PSR through the digital synthesis of spatially overlapping and much smaller effective pixels in each color channel. Furthermore, in D-PSR the pixel-level spectral cross-talk of a Bayer CFA is compensated by digital de-multiplexing. Compared to holographic color imaging using sequential multi-wavelength illumination, this new approach takes 3-fold less number of raw holograms/measurements while also achieving a very similar color imaging performance. D-PSR can be broadly used for high-resolution holographic color imaging and microscopy applications, where wavelength-multiplexing is desired.

## Additional Information

**How to cite this article**: Wu, Y. *et al*. Demosaiced pixel super-resolution for multiplexed holographic color imaging. *Sci. Rep*. **6**, 28601; doi: 10.1038/srep28601 (2016).

## Supplementary Material

Supplementary Information

## Figures and Tables

**Table 1:** Calibrated cross-talk matrix of our CMOS imager chip (Sony IMX81) at two sets of multiplexed wavelengths.

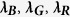	470 nm, 527 nm, 624 nm	471 nm, 532 nm, 633 nm
*W*	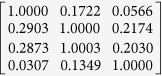	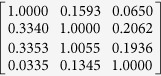

## References

[b1] Ozcan A. Mobile phones democratize and cultivate next-generation imaging, diagnostics and measurement tools. Lab. Chip 14, 3187–3194 (2014). 24647550 10.1039/c4lc00010b PMC4117730

[b2] Greenbaum A. . Wide-field computational imaging of pathology slides using lens-free on-chip microscopy. Sci. Transl. Med. 6, 267ra175–267ra175 (2014). 10.1126/scitranslmed.3009850 25520396

[b3] Brady D. J., Choi K., Marks D. L., Horisaki R. & Lim S. Compressive holography. Opt. Express 17, 13040–13049 (2009). 19654708 10.1364/oe.17.013040

[b4] Hahn J., Lim S., Choi K., Horisaki R. & Brady D. J. Video-rate compressive holographic microscopic tomography. Opt. Express 19, 7289–7298 (2011). 21503040 10.1364/OE.19.007289

[b5] Rivenson Y., Stern A. & Javidi B. Compressive fresnel holography. J. Disp. Technol 6, 506–509 (2010).

[b6] Rivenson Y., Stern A. & Javidi B. Overview of compressive sensing techniques applied in holography. Appl. Opt. 52, A423–A432 (2013). 23292420 10.1364/AO.52.00A423

[b7] Brooker G. . In-line FINCH super resolution digital holographic fluorescence microscopy using a high efficiency transmission liquid crystal GRIN lens. Opt. Lett. 38, 5264–5267 (2013). 24322233 10.1364/OL.38.005264 PMC3988313

[b8] Kanka M., Riesenberg R. & Kreuzer H. J. Reconstruction of high-resolution holographic microscopic images. Opt. Lett. 34, 1162–1164 (2009). 19370104 10.1364/ol.34.001162

[b9] Kashter Y., Rivenson Y., Stern A. & Rosen J. Sparse synthetic aperture with Fresnel elements (S-SAFE) using digital incoherent holograms. Opt. Express 23, 20941–20960 (2015). 26367947 10.1364/OE.23.020941 PMC4646520

[b10] Waller L., Tsang M., Ponda S., Yang S. Y. & Barbastathis G. Phase and amplitude imaging from noisy images by Kalman filtering. Opt. Express 19, 2805–2814 (2011). 21369102 10.1364/OE.19.002805

[b11] Memmolo P. . Automatic focusing in digital holography and its application to stretched holograms. Opt. Lett. 36, 1945–1947 (2011). 21593944 10.1364/OL.36.001945

[b12] Bianco V. . Optofluidic holographic microscopy with custom field of view (FoV) using a linear array detector. Lab. Chip 15, 2117–2124 (2015). 25832808 10.1039/c5lc00143a

[b13] Edwards C. . Diffraction phase microscopy: monitoring nanoscale dynamics in materials science. Appl. Opt. 53, G33–G43 (2014). 25322136 10.1364/AO.53.000G33

[b14] Kamilov U. S. . Learning approach to optical tomography. Optica 2, 517–522 (2015).

[b15] Laporte G. P., Stasio N., Sheppard C. J. & Psaltis D. Resolution enhancement in nonlinear scanning microscopy through post-detection digital computation. Optica 1, 455–460 (2014).

[b16] Girshovitz P. & Shaked N. T. Doubling the field of view in off-axis low-coherence interferometric imaging. Light Sci. Appl. 3, e151, doi: 10.1038/lsa.2014.32 (2014).

[b17] Tian L. & Waller L. 3D intensity and phase imaging from light field measurements in an LED array microscope. Optica 2, 104–111 (2015).

[b18] Wei Q. . On-chip cytometry using plasmonic nanoparticle enhanced lensfree holography. Sci. Rep. 3, doi: 10.1038/srep01699 (2013). PMC3632884 23608952

[b19] Su T.-W. . Sperm trajectories form chiral ribbons. Sci. Rep. 3, doi: 10.1038/srep01664 (2013). PMC3630328 23588811

[b20] McLeod E. . High-throughput and label-free single nanoparticle sizing based on time-resolved on-chip microscopy. ACS Nano 9, 3265–3273 (2015). 25688665 10.1021/acsnano.5b00388

[b21] Rivenson Y., Stern A. & Javidi B. Improved depth resolution by single-exposure in-line compressive holography. Appl. Opt. 52, A223–A231 (2013). 23292398 10.1364/AO.52.00A223

[b22] Rosen J. & Brooker G. Non-scanning motionless fluorescence three-dimensional holographic microscopy. Nat. Photonics 2, 190–195 (2008).

[b23] Stern A. & Javidi B. Theoretical analysis of three-dimensional imaging and recognition of micro-organisms with a single-exposure on-line holographic microscope. J. Opt. Soc. Am. A. 24, 163–168 (2007). 10.1364/josaa.24.000163 17164854

[b24] Barsi C., Wan W. & Fleischer J. W. Imaging through nonlinear media using digital holography. Nat. Photonics 3, 211–215 (2009).

[b25] Ferraro P. . Full color 3-D imaging by digital holography and removal of chromatic aberrations. J. Disp. Technol. 4, 97–100 (2008).

[b26] Kim T. . White-light diffraction tomography of unlabeled live cells. Nat. Photonics 8, 256–263 (2014).

[b27] Greenbaum A. . Increased space-bandwidth product in pixel super-resolved lensfree on-chip microscopy. Sci. Rep. 3, doi: 10.1038/srep01717 (2013).

[b28] Zhao J., Jiang H. & Di J. Recording and reconstruction of a color holographic image by using digital lensless Fourier transform holography. Opt. Express 16, 2514–2519 (2008). 18542331 10.1364/oe.16.002514

[b29] Göröcs Z., Kiss M., Tóth V., Orzó L. & Tokés S. Multicolor digital holographic microscope (DHM) for biological purposes . In *“BiOS”* 75681P–10, doi: 10.1117/12.841962 (International Society for Optics and Photonics, 2010).

[b30] Göröcs Z., Orzó L., Kiss M., Tóth V. & Tőkés S. In-line color digital holographic microscope for water quality measurements. in “Laser Applications in Life Sciences 2010” 737614–10, doi: 10.1117/12.871098 (International Society for Optics and Photonics, 2010).

[b31] Ito Y. . Four-wavelength color digital holography. J. Disp. Technol. 8, 570–576 (2012).

[b32] Greenbaum A., Feizi A., Akbari N. & Ozcan A. Wide-field computational color imaging using pixel super-resolved on-chip microscopy. Opt. Express 21, 12469–12483 (2013). 23736466 10.1364/OE.21.012469 PMC3686357

[b33] Kiss M. Z. . Special multicolor illumination and numerical tilt correction in volumetric digital holographic microscopy. Opt. Express 22, 7559–7573 (2014). 24718130 10.1364/OE.22.007559

[b34] Luo W., Greenbaum A., Zhang Y. & Ozcan A. Synthetic aperture-based on-chip microscopy. Light Sci. Appl. 4, e261, doi: 10.1038/lsa.2015.34 (2015).

[b35] Ramanath R., Snyder W. E., Bilbro G. L. & Sander W. A. Demosaicking methods for Bayer color arrays. J. Electron. Imaging 11, 306–315 (2002).

[b36] Lu W. & Tan Y.-P. Color filter array demosaicking: new method and performance measures. IEEE Trans. Image Process. 12, 1194–1210 (2003). 18237887 10.1109/TIP.2003.816004

[b37] Malvar H. S., He L. & Cutler R. High-quality linear interpolation for demosaicing of Bayer-patterned color images. in “*IEEE International Conference on Acoustics, Speech, and Signal Processing, 2004. Proceedings. (ICASSP ’04)”*, doi: 10.1109/ICASSP.2004.1326587 (2004).

[b38] Hirakawa K. & Parks T. W. Adaptive homogeneity-directed demosaicing algorithm. IEEE Trans. Image Process . 14, 360–369 (2005). 15762333 10.1109/tip.2004.838691

[b39] Farsiu S., Elad M. & Milanfar P. Multiframe demosaicing and super-resolution of color images. IEEE Trans. Image Process . 15, 141–159 (2006). 16435545 10.1109/tip.2005.860336

[b40] Hardie R. C., Barnard K. J., Bognar J. G., Armstrong E. E. & Watson E. A. High-resolution image reconstruction from a sequence of rotated and translated frames and its application to an infrared imaging system. Opt. Eng. 37, 247–260 (1998).

[b41] Bishara W., Su T.-W., Coskun A. F. & Ozcan A. Lensfree on-chip microscopy over a wide field-of-view using pixel super-resolution. Opt. Express 18, 11181–11191 (2010). 20588977 10.1364/OE.18.011181 PMC2898729

[b42] Greenbaum A. . Imaging without lenses: achievements and remaining challenges of wide-field on-chip microscopy. Nat. Methods 9, 889–895 (2012). 22936170 10.1038/nmeth.2114 PMC3477589

[b43] McLeod E., Luo W., Mudanyali O., Greenbaum A. & Ozcan A. Toward giga-pixel nanoscopy on a chip: a computational wide-field look at the nano-scale without the use of lenses. Lab. Chip 13, 2028–2035 (2013). 23592185 10.1039/c3lc50222h PMC3813829

[b44] Greenbaum A. & Ozcan A. Maskless imaging of dense samples using pixel super-resolution based multi-height lensfree on-chip microscopy. Opt. Express 20, 3129–3143 (2012). 22330550 10.1364/OE.20.003129 PMC3364049

[b45] Luo W., Zhang Y., Feizi A., Gorocs Z. & Ozcan A. Pixel super-resolution using wavelength scanning. Light Sci. Appl. 5, e16058, doi: 10.1038/lsa.2016.58 (2016). PMC6059953 30167157

[b46] Mudanyali O. . Compact, light-weight and cost-effective microscope based on lensless incoherent holography for telemedicine applications. Lab. Chip 10, 1417–1428 (2010). 20401422 10.1039/c000453g PMC2902728

[b47] Coskun A. F., Sencan I., Su T.-W. & Ozcan A. Lensless wide-field fluorescent imaging on a chip using compressive decoding of sparse objects. Opt. Express 18, 10510–10523 (2010). 20588904 10.1364/OE.18.010510 PMC2898750

[b48] Sencan I., Coskun A. F., Sikora U. & Ozcan A. Spectral demultiplexing in holographic and fluorescent on-chip microscopy. Sci. Rep. 4, doi: 10.1038/srep03760 (2014). PMC3895906 24441627

[b49] Goodman J. W. Introduction to Fourier optics (3rd Edition) 55–61 (Roberts and Company Publishers, 2005).

[b50] Teague M. R. Deterministic phase retrieval: a Green’s function solution. J. Opt. Soc. Am. 73, 1434–1441 (1983).

[b51] Zhang X. & Brainard D. H. Estimation of saturated pixel values in digital color imaging. J. Opt. Soc. Am. A. 21, 2301–2310 (2004). 10.1364/josaa.21.002301 PMC1815481 15603065

[b52] Dillon W. R. & Goldstein M. Multivariate analysis: Methods and applications . (Wiley New York, 1984).

[b53] Gunturk B. K., Glotzbach J., Altunbasak Y., Schafer R. W. & Mersereau R. M. Demosaicking: color filter array interpolation. IEEE Signal Process. Mag. 22, 44–54 (2005).

[b54] Bishara W., Zhu H. & Ozcan A. Holographic opto-fluidic microscopy. Opt. Express 18, 27499–27510 (2010). 21197025 10.1364/OE.18.027499 PMC3057885

