# Supplementary material for: Demosaiced pixel super-resolution for multiplexed holographic color imaging
Source: Sci Rep. 2016 Jun 29;6:28601. doi: 10.1038/srep28601 (PMC4926095; doi:10.1038/srep28601)
Supplement: Supplementary Information [file srep28601-s1.pdf]

Supplementary information for

Demosaiced pixel super-resolution for multiplexed holographic color imaging

Authors: Yichen Wu[1,2,3], Yibo Zhang[1,2,3], Wei Luo[1,2,3], Aydogan Ozcan[1,2,3,4,*]

Affiliations:

[1]Electrical Engineering Department, University of California, Los Angeles, CA, 90095, USA.

[2]Bioengineering Department, University of California, Los Angeles, CA, 90095, USA.

[3]California NanoSystems Institute (CNSI), University of California, Los Angeles, CA, 90095, USA.

[4]Department of Surgery, David Geffen School of Medicine, University of California, Los Angeles, CA, 90095, USA.

[*]Correspondence: Prof. Aydogan Ozcan
E-mail: ozcan@ucla.edu
Address: 420 Westwood Plaza, Engr. IV 68-119, UCLA, Los Angeles, CA 90095, USA
Tel: +1(310)825-0915
Fax: +1(310)206-4685

Authors' email addresses:

Yichen Wu: wuyichen@ucla.edu
Yibo Zhang: zybmax@g.ucla.edu
Wei Luo: luow@ucla.edu
Aydogan Ozcan: ozcan@ucla.edu

Optimization of the choice of illumination wavelengths in D-PSR

We analyzed the optimal multi-wavelength illumination choice based on the spectral characteristics of the Bayer CMOS imager chip (Sony IMX85) using a brute force search. We assumed that the main sources of de-multiplexing error on a single pixel come from: (1) thermal noise of the sensor, and (2) quantization noise. We should note that if the three multiplexed wavelengths are chosen to be too close to each other, the cross-talk among channels will be significant and the de-multiplexing matrix will be almost singular, causing any source of error (due to thermal noise and quantization noise) to be significantly amplified.

As detailed in the flow-chart shown in Supplementary Fig. S3, we did a brute force search, where we scanned all the possible wavelength combinations from 400 nm to 700 nm at 1 nm step size and calculated a de-multiplexing error for each combination. For this calculation, we first assumed that the input signal for each channel follows a Gaussian distribution with a mean of 1 and a standard deviation of 0.2. Then, using the measured/calibrated sensor response of the Bayer CFA, we generated a cross-talk matrix and using that we calculated the Bayer channel intensities. Each channel intensity also included a thermal noise term (~3.5%) and was quantized with a bit depth of 10 bits. Next, using Equation (2) of the main text, we performed the de-multiplexing step for all these RGB intensity points in our Gaussian distribution, the results of which are compared to the input distribution to calculate an average error for each channel. The total error for a given combination of illumination wavelengths is taken as the maximum of these three mean errors arising from R, G and B channels. Based on this combinatorial search, the optimal illumination wavelength trio that has the smallest de-multiplexing error is found to be (456 nm, 570 nm, 651 nm). However, the gradient near this optimal point is actually quite small and therefore a similar level of de-multiplexing error can be practically achieved over a large

spectral range (see Supplementary Fig. S4). For example, a region with a maximum de-multiplexing error of 6% (1.7x of thermal noise level) is shown in Supplementary Fig. S4(a) and it spans more than ~50 nm for all three Bayer channels. In Supplementary Fig. S4(b), we also show the cross-sectional 1D-plots of the de-multiplexing error near the choice of multiplexed-illumination wavelengths that we used in our experiments (i.e., 471, 532, 633 nm), confirming that the change of the de-multiplexing error is rather small over a relatively large spectral range.

YUV color-space averaging method

In the main text, one of the comparisons to D-PSR technique is made using the YUV color-space averaging method. In this technique, the color information is retrieved from three low resolution holograms at R, G and B color channels, which are then back-propagated to the sample plane, combined and transformed into YUV color-space, and low-pass filtered by an averaging window size of e.g., 10 pixels on the U and V channels to get rid of twin-image related rainbow artifacts of holographic imaging. The high resolution (i.e., pixel super-resolved) Y channel, which requires the acquisition of N raw holograms (same as D-PSR), and the low resolution U and V channels, which require the acquisition of 3 raw holograms, are then fused in the YUV color-space, and finally converted into RGB space to get a color image of the specimen.

Supplementary Figures and Figure Legends

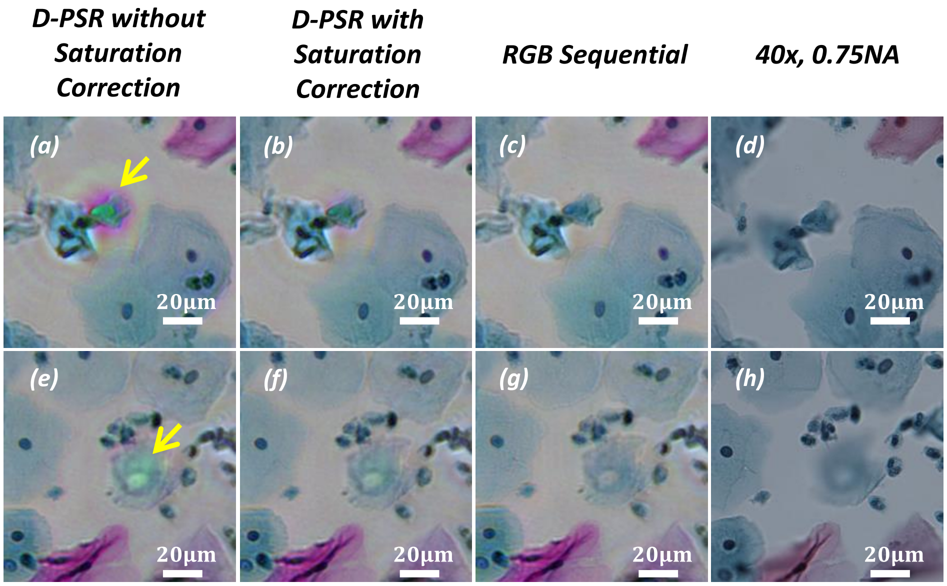

Supplementary Fig. S1. The impact of saturation correction in D-PSR images. D-PSR based image reconstruction (a, e) without and (b, f) with the saturation correction step. (c, g) The same regions of interest reconstructed using sequential RGB illumination, with 3-fold increased number of raw holograms. (d, h) Same samples imaged using a lens-based microscope (40x, 0.75NA). Some examples of prominent color artifacts are marked with yellow arrows in (a, e).

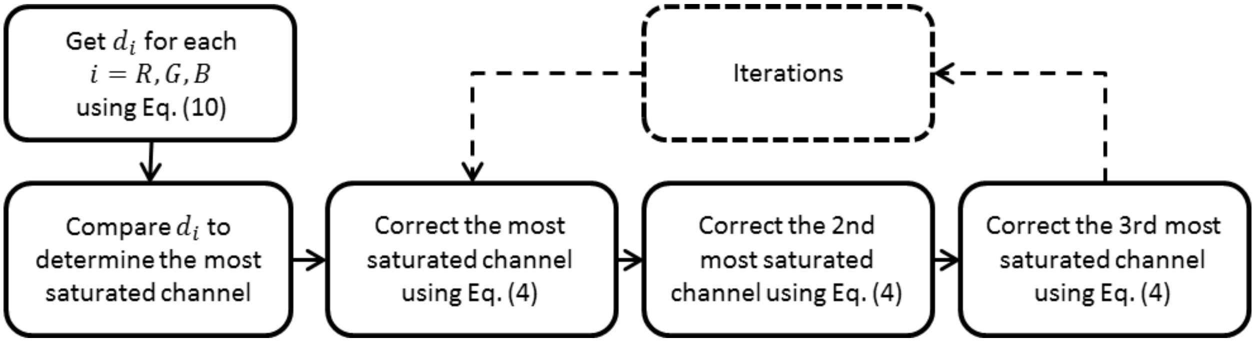

Supplementary Fig. S2. Flow chart for the saturation correction steps used in our D-PSR approach.

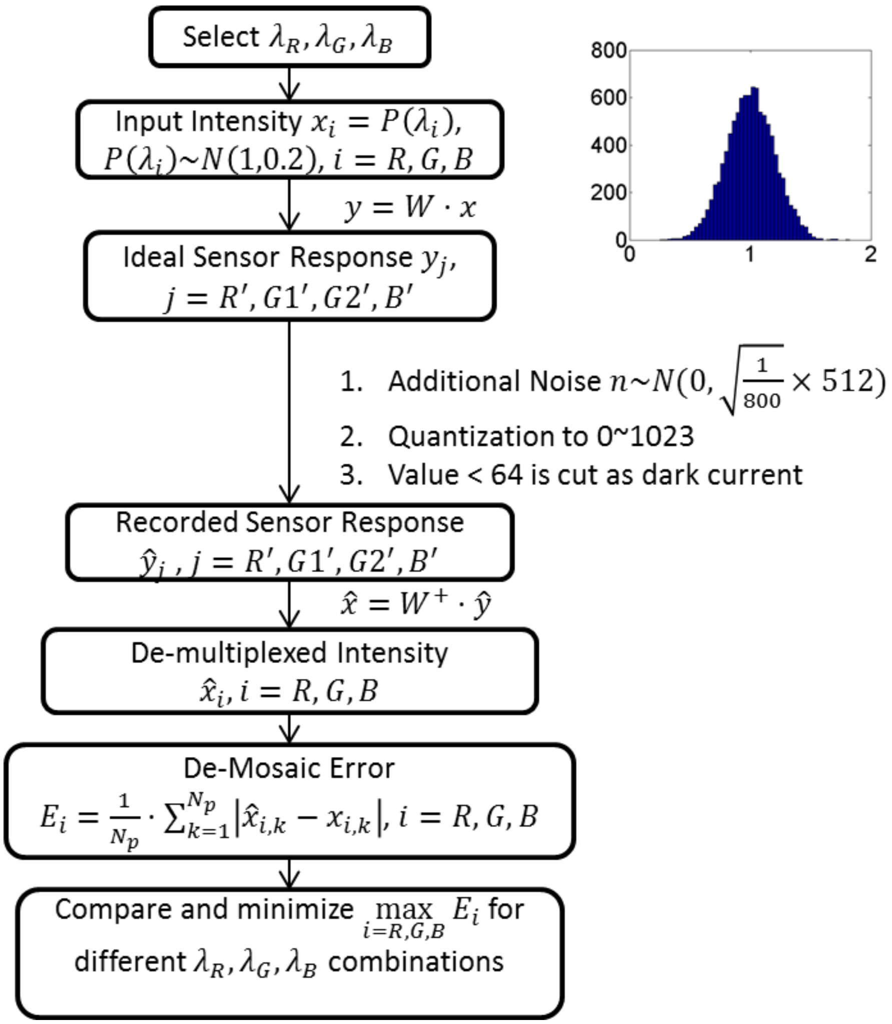

Supplementary Fig. S3. Flow-chart of de-multiplexing error calculation for different combinations of multiplexed illumination wavelengths in D-PSR.

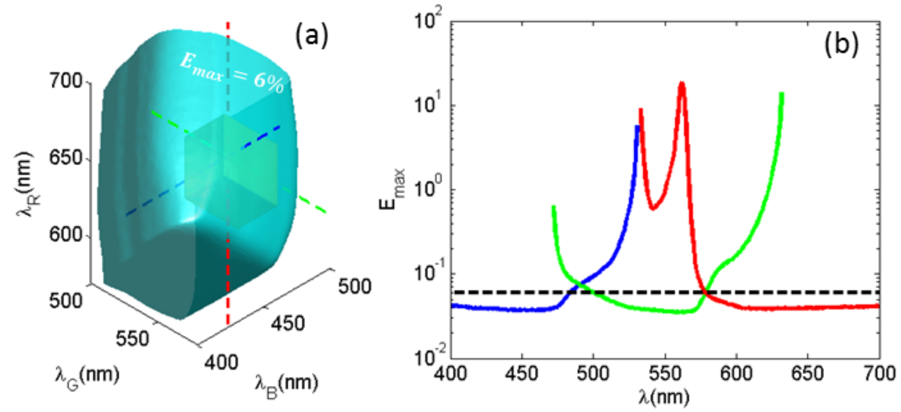

Supplementary Fig. S4. 3D search for optimization of the choice of illumination wavelengths in D-PSR. (a) The range of illumination wavelength combinations with a maximum de-multiplexing error of 6% (shown as the cyan surface) spans more than ~50 nm for all three color channels. A typical selection of red (~610-650 nm), green (~520-560 nm) and blue (~450-480 nm) illumination wavelengths (shown with the yellow cube) falls inside the 6% maximum error volume. (b) 1D cross sectional plots of the maximum de-multiplexing error, each of which passes through the point $(\lambda_B, \lambda_G, \lambda_R) = (471,532,633) nm$ in (a). The dashed black line indicates an error threshold of 6%.
